# Promoting recovery-oriented practice in mental health services: a quasi-experimental mixed-methods study

**DOI:** 10.1186/1471-244X-13-167

**Published:** 2013-06-13

**Authors:** Helen Gilburt, Mike Slade, Victoria Bird, Sheri Oduola, Tom KJ Craig

**Affiliations:** 1 King’s College London, Addictions Department, Institute of Psychiatry, London, UK; 2 King’s College London, Health Service and Population Research Department, Institute of Psychiatry, London, UK

**Keywords:** Recovery, Mental health, Health services, Implementation, Organizational change

## Abstract

**Background:**

Recovery has become an increasingly prominent concept in mental health policy internationally. However, there is a lack of guidance regarding organisational transformation towards a recovery orientation. This study evaluated the implementation of recovery-orientated practice through training across a system of mental health services.

**Methods:**

The intervention comprised four full-day workshops and an in-team half-day session on supporting recovery. It was offered to 383 staff in 22 multidisciplinary community and rehabilitation teams providing mental health services across two contiguous regions. A quasi-experimental design was used for evaluation, comparing behavioural intent with staff from a third contiguous region. Behavioural intent was rated by coding points of action on the care plans of a random sample of 700 patients (400 intervention, 300 control), before and three months after the intervention. Action points were coded for (a) focus of action, using predetermined categories of care; and (b) responsibility for action. Qualitative inquiry was used to explore staff understanding of recovery, implementation in services and the wider system, and the perceived impact of the intervention. Semi-structured interviews were conducted with 16 intervention group team leaders post-training and an inductive thematic analysis undertaken.

**Results:**

A total of 342 (89%) staff received the intervention. Care plans of patients in the intervention group had significantly more changes with evidence of change in the content of patient’s care plans (OR 10.94. 95% CI 7.01-17.07) and the attributed responsibility for the actions detailed (OR 2.95, 95% CI 1.68-5.18). Nine themes emerged from the qualitative analysis split into two superordinate categories. ‘Recovery, individual and practice’, describes the perception and provision of recovery orientated care by individuals and at a team level. It includes themes on care provision, the role of hope, language of recovery, ownership and multidisciplinarity. ‘Systemic implementation’, describes organizational implementation and includes themes on hierarchy and role definition, training approaches, measures of recovery and resources.

**Conclusions:**

Training can provide an important mechanism for instigating change in promoting recovery-orientated practice. However, the challenge of systemically implementing recovery approaches requires further consideration of the conceptual elements of recovery, its measurement, and maximising and demonstrating organizational commitment.

## Background

The notion of recovery is becoming ever more prominent in mental health treatment. Originating from consumer perspectives challenging traditional beliefs about course of illness and treatment, it has come to be widely conceptualised as a process of building a meaningful and satisfying life, as defined by the person themselves, whether or not they are experiencing ongoing or recurring symptoms or problems associated with illness [1]. The strength of the concept has resulted in recovery being identified as a guiding principle in policies defining the delivery of mental health care provision in a number of countries including the USA, Canada, New Zealand and most recently the UK [2-5] Despite this, recovery and its key components are under continuous debate and the idea of recovery remains controversial.

Much of the contention surrounding recovery has resulted from its inherently individualistic nature. The approach has been perceived as challenging professional expertise, and tensions have arisen in areas such as working in the best interests of patients and the provision of evidence-based care [6]. On a service provider level, recovery can present particular challenges in accommodating self-determination and choice along with the public protection expectations on the system. Researchers have addressed these tensions by developing conceptual frameworks for personal recovery. These draw together the seemingly disparate concepts or components into models which describe the key characteristics and processes encompassing recovery [7]. Taking all of these factors into account, proponents suggest that successful implementation of recovery requires a service transformation towards mental health systems with a different values base [8]. These challenges may limit implementation. In the UK the growth of recovery-orientated services has been slow and patchy. Despite this, providers are now seeking to integrate the developing evidence base on recovery-orientated care to transform their own services.

One approach to supporting practice change has been through training. For example, training programmes underpin much of the system of knowledge transfer across the UK healthcare system. Programmes can be standardised, used across large populations, and allow measurable outputs to be embedded. Studies in the USA [9] and Australia [10] provide some evidence that structured training on critical components of recovery can increase both knowledge and pro-recovery attitudes. A growing number of recovery training programmes, including some that have been granted national accreditation, have been developed in the UK. However, empirical evidence of a positive impact is limited. Exploration of this area may provide valuable insight into how best to approach the implementation of a recovery orientation, and offer a better understanding of the barriers and facilitators of change in practice across wider healthcare systems.

Drawing on a previous pilot study and utilising the training programme developed [11], we aimed to implement a programme of recovery training for mental health staff working in services across two regions of London and compare the effects with a third region in which no training had taken place. Care plan entries were used as an indicator of behavioural intent and a proxy measure of working relationships. It is hypothesised that training would lead to an increase in diversity of care and a decrease in the proportion of staff-led care both of which may indicate an increased orientation towards recovery. Qualitative interviews were used to investigate implementation influences at individual and team levels.

## Methods

The study utilised a mixed methods quasi-experimental design comprising a quantitative care plan audit and qualitative interviews with participating staff members. Ethical approval was obtained from King’s College London Research Ethics Committee, and local permission was obtained from South London and Maudsley NHS Foundation Trust.

### Site and respondent selection

The participating service provider provides a full range of mental health services including all community-based and in-patient rehabilitation adult mental health teams for the inner-city London Boroughs of Lambeth (population 303,100), Southwark (population 288,300) and Lewisham (inner city, population 275,900) [12]. Lambeth and Southwark were non-randomly allocated to the intervention arm and Lewisham to the control arm. The three Boroughs are comparable in the services they provide and staffing. No identifiable patient-level information was collected, but all the demographics of each region are similar – high levels of deprivation, caseloads across all community teams likely to comprise approximately 70% psychosis and 35%-40% minority ethnic (especially African and African-Caribbean).

### Intervention

The intervention comprised four full-day workshops in a classroom setting, followed by an in-team half day session. The content was developed by the research team and project steering group comprising health service researchers, clinicians, service users and carers, with support from the health provider’s training department. Training took place between January 2008 and January 2009, and attendance was mandatory. Day 1 comprised an introduction to recovery, and reflection on the different elements that constitute a recovery approach. Days 2 and 3 utilised an established recovery training package called *Psychosis revisited - a psychosocial approach to recovery*[13]. Day 4 covered a range of topics: assessment and care planning from service users’ perspectives; social inclusion/vocational activities from a social work perspective; carer perspectives on recovery; spirituality and reflection on fundamental issues around personal values and beliefs, strengths based approaches, and the role of hope. The diversity of trainers (with both professional expertise and lived experience) aimed to model partnership working, maximize experiential learning and provide individual examples of recovery and recovery-orientated practice. Trainers attended a supervisory group to ensure consistency and receive personal support. The workshops and process of delivery aimed to develop knowledge and subsequently link theory to practice addressing issues of implementation at each stage. Each workshop ran twice in the same month to maximize attendance. Following these workshops, a half-day consolidation meeting with individual participating teams was held, to support team members to reflect on the active ingredients of the training, how these were being used in practice in their team, and how the concept of recovery would be sustained in individual teams.

### Data collection and analysis

An audit of care plans on the local clinical information system was undertaken at the baseline (start of training) and three-months post-training (15 months after baseline). The electronic records of a random sample of 400 patients stratified by participating teams were drawn from the caseloads of staff who had attended the training and 300 from staff in equivalent teams in the control borough were selected. Each action point was coded according to the topic of action using a pre-determined list of categories, and who would take responsibility for the action: “Staff”, “Service user” or “Carer”, alone or jointly. Data were analyzed using STATA version 11. Analyses were conducted to examine two outcomes, 1) change in care plan topics resulting from the removal or addition of topics; and 2) change in responsibility of action. Since individual care plans comprised a number of action points each related to a different topic of care, the impact of the training intervention on these outcomes was explored through random effects logistic regression taking account of clustering by patient.

Team leaders from each participating service who had attended at least one day of the training were invited to participate in a semi-structured interview. Written informed consent was obtained from those who agreed. The interview topic guide was developed in collaboration with a group of experts and explored team leaders’ understanding of recovery, implementation within the service and the wider Trust, and the perceived impact of the training on their individual practice and that of their wider team. Interviews were conducted 3 months post-training by an independent researcher, audio-taped and transcribed verbatim. The transcripts were coded by a member of the research team using NVIVO 7. The interview guide questions served as a provisional starting list of a priori codes by which to analyse the data. The coding frame was then elaborated and modified as new themes and subthemes emerged in the course of the analysis. The developing coding frame was discussed amongst the research team – a service user researcher, psychiatrist, clinical psychologist and psychiatric nurse, until a consensus was reached.

## Results

### Training attendance

Twenty two mental health teams participated in the intervention, comprising early intervention for psychosis (n = 2), community mental health (n = 5), in-patient rehabilitation services (n = 3), assertive outreach (n = 2), and continuing care teams (n = 10). This represented the full range of non-crisis mental health teams operating in the two Boroughs.

The teams comprised a total of 428 mental health professionals at the start of the study. Of these 383 (91%) registered on the training programme, including 193 (50%) care coordinators (predominantly nursing staff), 81 (21%) support workers, 22 (6%) team leaders and 87 (23%) staff from other professional groups (psychologists, psychiatrists, approved social workers, chaplains and vocational workers). Non-registrants included the night-staff from one rehabilitation ward and a number of staff whose role had changed or had moved teams prior to the start of the training and were no longer eligible.

Of the 383 professionals who registered, 342 (89%) staff attended at least one training session, and 190 staff (48%) attended all four classroom-based workshops. There was a gradual decline in attendance for consecutive workshops from 272 (69%) in the first workshop, 261 (66%) for workshop 2 and 3 combined and 197 (50%) for workshop 4. Staff turnover during the training programme was 21%, with 46 new staff joining participating teams and 41 staff leaving those teams.

### Care plan audit

Care plans for 700 patients (400 intervention, 300 control) were reviewed. A total of 673 paired pre- and post-training care plans were available, with 27 (15 intervention, 12 control) excluded due to missing data. The care plans contained 3,526 distinct action points at baseline (1,870 intervention, 1,656 control), and 3,629 at follow up (1,939 intervention, 1,690 control). Staff took sole responsibility for the majority of actions listed in care plans at baseline and follow up, as shown in Table 1.

**Table 1:** Number of action points at baseline and follow up by responsibility for action

	**Baseline (%)**		**Follow up (%)**	
	**Control**	**Intervention**	**Control**	**Intervention**
Staff	1090 (65.8)	1399 (74.8)	1106 (65.4)	1382 (71.3)
Service user	130 (7.9)	157 (8.4)	140 (8.3)	167 (8.6)
Staff and service user	413 (24.9)	312 (16.7)	416 (24.6)	385 (19.9)
Staff and carer	12 (0.7)	1 (0.1)	13 (0.8)	3 (0.2)
Service user and carer	8 (0.5)	1 (0.1)	11 (0.7)	1 (0.1)
Carer	3 (0.2)	0	4 (0.7)	1 (0.1)
**Total**	**1656**	**1870**	**1690**	**1939**

The topics present in the care plans related predominantly to care plan review meetings, medication and relapse prevention, as shown in Table 2.

**Table 2:** Topics addressed in baseline care plans (n = 673)

	**Number (%) of care plans**	
**Topic**	**Control (*n = 296)***	**Intervention (*n = 377)***
Care plan review meeting	296 (100)	326 (86.5)
Relapse prevention	293 (99.0)	325 (86.2)
Medication	240 (81.1)	263 (69.8)
Physical health	212 (71.6)	180 (47.7)
Activities of daily living skills #	151 (51.0)	156 (41.4)
Accommodation	101 (34.1)	66 (17.5)
Social needs	76 (25.7)	116 (30.8)
Financial	66 (22.3)	74 (19.6)
Emotional support	65 (22.0)	165 (43.8)
Employment	51 (17.2)	44 (11.7)
Healthy lifestyle	40 (13.5)	93 (24.7)
Education	37 (12.5)	30 (8.0)
Carer support	28 (9.5)	32 (8.5)

#, e.g. self-care, housekeeping, shopping, cooking, use of public transport.

A total of 46 changes were made to care plans in the comparison group and 573 changes in the intervention group, as shown in Table 3.

**Table 3:** Changes to care plan action points by topic at follow up

	**% of care plans with action points removed**		**% of care plans with new action points**	
	**Control**	**Intervention**	**Control**	**Intervention**
Care plan review meeting	0	15.6	0	11.9
Relapse prevention	0	12.2	0	9.3
Medication	0	2.9	1.7	8.0
Physical health	0	5.3	2.0	8.5
Activities of daily living skills	0	5.0	1.4	8.2
Accommodation	0.3	2.9	1.0	3.2
Social needs	0.7	4.8	2.4	6.9
Financial	0.3	3.7	0.3	5.3
Emotional support	0	4.8	1.0	9.8
Employment	0.3	1.9	1.4	4.0
Healthy lifestyle	0	4.0	1.4	6.6
Education	0.3	1.1	1.0	2.4
Carer support	0	2.7	0	1.1
**Total no of changes**	**6**	**252**	**40**	**321**

Patients in the intervention group had increased odds of having a change in the topics covered in their care plan at follow up compared with the control group OR = 10.94 (95% CI 7.01-17.07). This represents both the addition and removal of topics to the care plan. There is no clear trend in particular topics of care being removed or added, for example, 15.6% of care plans had the entry related to care plan review removed, whilst 11.9% had an entry in the same category added in the intervention group.

The attributed responsibility was changed for 33 action points in the control group and 93 in the intervention group, as shown in Table 4.

**Table 4:** Changed, removed or added action points at follow up distributed by attributed responsibility for action

	**Control (%)**			**Intervention (%)**		
	**Action points changed**	**Action points removed**	**New action points**	**Action points changed**	**Action points removed**	**New action points**
Staff	11 (33)	4 (66)	22 (55)	23 (25)	222 (88)	238 (74)
Service user	7 (21)	0	6 (15)	12 (13)	7 (3)	19 (6)
Staff and service user	11 (33)	2 (33)	11 (28)	54 (58)	22 (9)	63 (20)
Staff and carer	1 (3)	0	0	2 (2)	0	1
Service user and carer	3 (9)	0	0	1 (1)	1	0
Carer	0	0	1 (2)	1 (1)	0	0
**Total**	**33**	**6**	**40**	**93**	**252**	**321**

Patients in the intervention group had increased odds of the responsibility for actions being changed in existing topics covered in their care plan at follow up compared with the comparison group OR = 2.95 (95% CI 1.68-5.18). The majority of these changes related to whether staff took sole responsibility for actions (33% control, 25% intervention) or shared responsibility with service users (33% control, 58% intervention). This trend is also reflected in the topics that had been removed or added to care plans at follow up with the majority of changes relating to topics in which responsibility for action was attributed solely to staff or to staff in collaboration with patients.

### Qualitative evaluation

Two superordinate themes emerged from the 16 team leader interviews: Recovery, individuals and practice (with five themes) and Systemic implementation (four themes).

### Recovery, individuals and practice

This theme describes the perception and provision of recovery orientated care by individuals and at a team level.

#### Care provision

All participants identified a range of interventions including medication, symptom management, and psychological therapies, in addition to practical elements such as meaningful activity, training and stress management that comprise a recovery approach. There was a strong emphasis on social inclusion interventions as integral to a recovery focus. Some identified that the care provided needed to be holistic, taking into account the emotional, spiritual, social, physical and realms which impact on patients’ quality of life including relationships. Training had led to staff considering wider areas of care to a greater extent with a consequent move from maintenance to improvement. Qualities required to deliver recovery focused care included the ability to be caring, helping, supporting, respectful and open. A minority highlighted a conceptual element to recovery orientated care involving the way you looked at people and thought about things.

#### Hope

Hope was highlighted as central to providing recovery-orientated care. The majority conceptualised hope as seeking positive change, while some participants working with people with long term severe mental illness felt it could also encompass a lack of change for the worse.

#40 “*Hope means to me, I think for my clients we hope that people do change, and it is helpful you know in terms of one of the workshops I went on, it was about not giving up on people and introducing hope…*”

Hope was seen as a universally positive value and integral to mental health work, although many reported low levels of morale and hope amongst staff within their services. The majority of participants talked about the role of hope for staff, a minority highlighting its role for patients. Hope involved valuing patients as individuals and having belief in patients. Many participants found it difficult to identify how it could be practically implemented. Those who did suggested that short term it was useful in reaching goals and outcome specific tasks through encouragement. Some participants were wary of the use of hope beyond this, engendering unachievable ‘blue sky’ goals.

#### Language

There was much confusion about what ‘recovery’ meant and this impacted directly on participants perceptions of what recovery-orientated practice comprised. Participants noted that many members of staff believed they ‘already did recovery’.

#29 “*…there were comments that there is no theoretical base in the recovery approach, it is an approach it is not a model, there is no clear definition of recovery or there are several definitions, lots of comments what is the point of the training when we are already doing this.*”

The provision of practical care focused on social inclusion, such as help with employment, was seen to support this stance. The word recovery was strongly associated with the verb ‘to recover’ and recovery was seen by the majority as a linear journey with a start and end point. A small number of participants identified the word recovery as inherent in a number of other Trust initiatives, such as ‘Support Time Recovery Workers’ and used these as examples of recovery practice. Language was also identified as an important component of recovery approaches. Two participants noted that with a new model, there was new language. Use of this new vocabulary could identify the unique nature of recovery, while some of the current language in use was seen as not being recovery focused. The training led some staff to reflect on their own use of language.

#### Ownership

Recovery was largely framed as something that staff do, with staff being the primary agents of change. Staff took ownership of recovery, its meaning and implementation, with care provision mediated by their perceptions of recovery.

#37 “*…so sometimes we can improve their life with social inclusion working towards vocational activities, helping them to build up their lives in terms of psychosocial education activities, education and, also we give them, very fortunate here, we’ve got psychology*”

Few interviewees noted the role of service users. Of those that did, service user involvement was seen as being part of the approach, with involvement ranging from being ‘included’, ‘being part of recovery’ to in one instance ‘taking charge’. In examples when involvement was identified as important, staff were identified as facilitators of patient-led care, where the ability to work in partnership and enable patients to think about recovery were important.

#### Multidisciplinary

Multidisciplinary working was highlighted as important in the provision of recovery focused care. Several interviewees highlighted different schools of thought and broad principles which predominate in, and to some extent define, different professional groups. Interviewees stated that purveyors of the medical model were least likely to be recovery-focused while those adhering to social models of illness were most likely. Doctors were seen as least recovery–focused and social workers as most.

#40 “*I think traditionally it has always been a very medically based model, …I think social workers slightly have the edge in terms of using a recovery model using a social care model in terms of helping clients, I think nurses traditionally take on board what doctors say and sort of the care plan has traditionally been directed by consultants for example…*”

It was clear that levels of hierarchy existed in many of the services. Where doctors were not on-board with the training and recovery in general, they could act as a barrier. Conversely, doctors who promoted the approach acted as role models. Despite a focus on professions, several interviewees noted that recovery had to be multidisciplinary, with professions learning from each other, and all clinical staff needing to adopt the approach for it to work effectively. While this theme focuses largely on the role of professions, some participants noted that although teams had been generally positive about recovery, there were some individuals who were resistant to change. Despite training and development of practice, these staff were unlikely to change their views and ways of working.

#48 “*So I think it’s down to managers to ensure that they have embraced the model. And that it’s cascaded down and that staff also embrace it and apply it within their practise. But I don’t think that’s something that [the Trust] can do. I think that’s the individual’s responsibility. Because you teach someone as much as you wanna teach them, if they don’t wanna apply it, they won’t.*”

It was on the individual level that interviewees reported changes since the training. They had observed that staff were beginning to consider wider and more holistic care provision, such as taking into consideration spirituality and looking at options such as activity and vocation. There was a move from a focus on maintenance towards improvement, looking forward beyond crisis management to what happens next, including when patients left their care and potentially the care of the mental health services altogether. Some staff had been adopting new recovery-related terminology and reconsidering the language commonly associated with predominating ideologies.

### Systemic implementation

Interviewees highlighted a number of areas which had created barriers to a more substantial and wider felt impact across those services involved. These pertained more widely to structural elements of care provision and are demonstrated in the last four themes.

#### Hierarchy and role definition

Interviewees made it clear that in terms of practice, they exist not just as individual practitioners but within services, and the wider system of an NHS Trust. The relationship is described as hierarchical with Trusts determining the role and practice of services. A number of participants highlighted the ‘needs of the service’ to meet these. Recovery orientated approaches were often seen as conflicting with the overarching roles of the service. The most prominent role in community teams was ‘moving people on’. It was described as having a single vision for patients and comprised entering services highly symptomatic with poor functioning and leaving with improved management and functioning.

#26 “*…we are about moving people through from very high support needs, people that have just moved in maybe to rehabilitation to residential services, to very low support and on into the community, so I guess we have always worked that model of moving people through a system…*”

Other roles included detention (inpatient services) and risk management. While roles were widely accepted, the accompanying policies, procedures and targets were identified as presenting often ideological and practical barriers to recovery orientated care provision. Participants suggested that in order for services to become recovery orientated, recovery would need to be embedded in the service’s role and to underpin everything it did. Two exceptions were assertive outreach and early intervention teams, both of which had clear identities and roles largely determined by the client group and specific model of care provision.

Recovery was identified by several participants as a Trust ‘initiative’. Despite recognition that the Trust was committed to recovery, there was a lack of clarity about what the Trust meant by recovery, how it related to other initiatives and Trust strategies, and in particular what this meant in terms of the role of services. This led some interviewees to suggest that a recovery approach was being implemented for political reasons, to meet government targets, as a tool for reducing costs, and like previous initiatives, may soon be de-prioritised. Interviewees highlighted a lack of communication and shared understanding between management and staff in services of the vision, and implications, for care provision.

#46 “*In terms of the commitment, I don’t know if everybody in [the Trust] is probably singing from the same sheet, I don’t think that that’s the case…*”

Interviewees suggested that without commitment from the Trust as a whole to address these issues, a recovery approach was likely to be unsustainable within services in the long-term.

#### Training approaches

The training was highly rated, with over half of interviewees favouring mandatory recovery training. Participants particularly valued input from service users and the chaplaincy. The former were described as real life examples of recovery with often long histories of severe mental illness, now delivering training. This input was particularly effective when their experiences were representative of the services’ client group. The chaplaincy was identified as highlighting the role of spirituality and different world views. However, a focus on practical elements such as social interventions led to widespread scepticism of a recovery approach as a repackaging of something they already did.

#40 “*I think we were doing it anyway the mental health teams, and in terms of care coordinators you do try and help clients access other services, you know become more independent, vocational and training and work needs.*”

The reflexive practice embedded in the training was valued highly by staff with strong agreement that this activity should be incorporated into overall practice. Some team leaders had implemented regular sessions to examine day to day practice, values and conceptions as a result of the training. The provision of training was seen as denoting the importance of the approach and emphasis by the Trust. However, recovery was seen as a process and it was suggested that training needed to be ongoing with systemic changes and support from the wider Trust to implement and sustain recovery approaches.

#### Measures of recovery

Measurement and measures of recovery were identified as important factors in implementation. Systematic measurement of impact was highlighted as demonstrating the priority of an intervention for the Trust and more widely as a means of improving the evidence base and legitimacy of the approach. Measures also provided a means of ensuring the approach was being used, and of encouraging and recognising good practice.

#35 “*…unless something is measured there’s no real inducement and encouragement to do it unfortunately…*”

Staff focused on staff-rated changes for patients, such as improved functioning levels, generating hope and how this could lead to tangible outcomes. Staff-related outcomes included changes in attitudes and team approaches. Three members of staff highlighted the importance of patient-identified and patient-rated outcomes.

#### Resources

The majority of interviewees identified resources as a key consideration in the implementation of recovery and providing recovery orientated care. Identified resource constraints included: expertise such as vocational workers and recovery champions; community resources such as appropriate placements and accommodation; engagement activities; training; but most importantly resources to cover staff numbers and time. The majority of interviewees believed that a recovery approach would require increased staff numbers and time, initially in attending training but also that recovery approaches would involve working more intensively and for longer periods with patients. These resources are governed by money.

#29 “*…you have to look at resources and how they impact or lack of resources, at times in an ideal world it is all well and good talking about a recovery approach and how we implement that but is, you know to look at and consider certainly resources and how that impacts on teams and then time and people’s workloads….*”

Almost half identified that the qualities possessed by individual staff were also important in the implementation and practice of recovery. Among the qualities highlighted were skills, experience, motivation, energy, flexibility, creativity, commitment, open-mindedness, a positive attitude, caring, and amenable to change. Some of these qualities were identified as characteristics that could be developed, while others as inherent in a person’s beliefs, values and personality.

## Discussion

The training programme had a positive impact, with evidence of change in both the content of patient’s care plans and the attributed responsibility for the actions detailed. However, the hypothesized changes towards diversification of care plan topic entries and collaborative responsibility for actions were not demonstrated. Evidence of change is also supported by the interviews with the team leaders. They identified changes in staff approaches to care and practice, including a greater consideration of holistic care provision, a move from focusing on maintenance to improved mental health and outcomes, and reflection on the use of language including utilization of new ‘recovery-related’ terminology. Staff were reported to be increasingly reflective about care provision, recovery approaches and practice with some teams incorporating reflective practice into supervision and team meetings. Despite this, over half of the team leaders interviewed stated that the training had little or no impact and in some cases, the training may have had a negative impact, reinforcing the belief that recovery was nothing different from what was being done already.

The translation of clinical interventions into routine practice has been identified as a key area of importance and in which the evidence base, particularly in mental health, is weak. Theoretical frameworks attempting to concepualise this process are underpinned by a recognition of different stages and mechanisms of change, and multiple foci of action [14-16]. A systematic review of behavioural change suggests that training is most effective in addressing the capabilities of individuals through imparting knowledge but less so in addressing motivation [14]. This may explain our finding that the training intervention was effective in raising staff awareness of recovery principles, and encouraging them to revisit and reconsider the content of care plans, but did not go so far as to focus subsequent action. Additional approaches focused on reinforcing motivations for implementing recovery-orientated care and environmental restructuring may increase effectiveness and address the issues of organizational support raised by team leaders.

### Communicating principles of recovery

Interviewees’ accounts often reflected a struggle to define recovery and its components. This resulted in a focus on specific elements, such as vocational training, or approaches like social work or patient-centred care being identified as demonstrative of ‘doing’ recovery, omitting the underlying philosophy of recovery-orientated practice. This confusion may detract from a clear pathway of change and the resulting lack of direction in the changes found in this study. It is reflective of the wider literature and a major criticism of the model by opponents [17]. These difficulties have been described as being a result of a lack of conceptual clarity and researchers of recent have sought to remedy this. The conceptual frameworks proposed by Farkas [18], Leamy [7] and Whitley and Drake [19] draw away from specific actions, instead focusing predominantly on values inherent in recovery approaches; characteristic processes and stages of recovery; and dimensions of recovery, respectively. Training and implementation programmes based on these frameworks may be more effective in integrating ideological and practical elements to provide a comprehensive understanding of recovery and a basis for translating theory into practice.

### Measuring implementation

Care plans provide an important measure of intent and action but our research suggests that this may have limitations in recording the implementation of recovery-orientated practice. Early stages of change associated with adoption of an intervention by individuals [16], such as re-contemplation of care for individuals and changes in values and relational approaches underpinning recovery-orientated practice may have been missed given the focus on actions. Furthermore, requirements of care planning and the formal nature of entries and language used may have also proved an additional barrier to recording changes in practice, particularly in relation to responsibility. A requirement for effective measures of recovery has been identified in the literature [20] and by interviewees in this study. Measures serve a number of uses, including validating the importance of an approach, benchmarking progress, and providing metrics for accreditation or recognition of success. Outcome measurement in health services is a policy priority in the UK [5]. However it has been suggested that limitations in the scope and context of current measures available makes measurement of recovery a challenge for services [21]. Further research in this area may prove important in developing measures which encompass the various characteristics, processes and stages of recovery while fulfilling the requirements of services and the wider system.

### Organisational considerations for implementation

This is one of the first studies to report on the implementation of recovery practice across a system of services. The training programme was undertaken with the support of the service provider involved, however the decreasing attendance throughout and interviewees’ responses questions the role of the wider system in implementing service level change. Perceived structural barriers such as defined service role, current policies and Trust commitment to recovery approaches were identified as providing sources of conflict with the staff role in delivering recovery-orientated care. Studies of programme implementation in health suggest that attention to organisational culture and climate are key to success [22]. A number of core cultural elements have been identified as important including organizational commitment [23,24], and a requirement for an organisation’s mission, policies, procedures, record-keeping and staffing to be consistent with recovery values in order for a programme to be successful [18]. Furthermore, although recovery practice itself need not be resource intensive, consideration of existing resources has been found to be important in supporting and maintaining change [23,25]. These views are very much supported by the interviewees in this study. Extending training programmes to wider staff and management may be one way of addressing these concerns but is likely to be insufficient without leadership, organizational culture change and enforcement through supervision [26].

### Strengths and limitations

The study of implementation is a relatively new field of enquiry. This study was conducted to reflect predominant training implementation practices in the UK. One of the strengths therefore is that it the findings can be related to the current practices of providers. Furthermore the use of a mixed methods design combining an overarching measure of impact with the experiences and insights of staff at the focus of the intervention provides important knowledge about of the process of implementation generalizable to other organisations. However, in not conducting a randomised controlled trial we were unable to control for differences between the control and intervention groups at baseline and the lack of blinding may have led to the introduction of bias. Additionally, the lack of sensitivity in the care plan audit to different stages of change may have reduced our ability to detect the full impact of the training.

## Conclusions

This study highlights some key issues in implementing the recovery model across mental health systems with implications for future development. Our results support the use of training approaches as a mechanism for knowledge transfer and facilitating implementation. However, there is a need to develop training better aligned with the emerging conceptual dimensions of recovery [7,19] and organisations should be cautious in relying on training programmes which alone are unlikely to be sufficient to create widespread and sustained change. The use of measures is important in supporting and evaluating implementation. Further research is required to develop measures of implementation that target different facets of change and the translation of this to patient care. Most importantly, implementation needs to move beyond the frontline workforce. Ensuring recovery-orientated practice is embedded in the core identity and role of mental health service providers, alongside developing an understanding of the process of change and broader systemic influences, will be crucial in supporting organizational transformation.

## Competing interests

The authors declare that they have no competing interests.

## Authors’ contributions

M.S. conceived of and designed the study. H.G and S.O collected the data. H.G, V.B and T.C conducted the analysis. H.G drafted the manuscript, with guidance and input throughout from S.O, M.S, V.B and T.C. T.C was project manager and M.S. was the chief investigator on the project grant. All authors read and approved the final manuscript.

## Pre-publication history

The pre-publication history for this paper can be accessed here:

http://www.biomedcentral.com/1471-244X/13/167/prepub
